# Supplementary material for: Label-free analysis of physiological hyaluronan size distribution with a solid-state nanopore sensor
Source: Nat Commun. 2018 Mar 12;9:1037. doi: 10.1038/s41467-018-03439-x (PMC5847568; doi:10.1038/s41467-018-03439-x)
Supplement: Supplementary file 1 — Supplementary Information [file 41467_2018_3439_MOESM1_ESM.pdf]

Supplementary Information

Label-free analysis of physiological hyaluronan size distribution with a solid-state nanopore sensor

Felipe Rivas, Osama K. Zahid, Heidi L. Reesink, Bridgette T. Peal, Alan J. Nixon, Paul L. DeAngelis,

Aleksander Skardal, Elaheh Rahbar, and Adam R. Hall

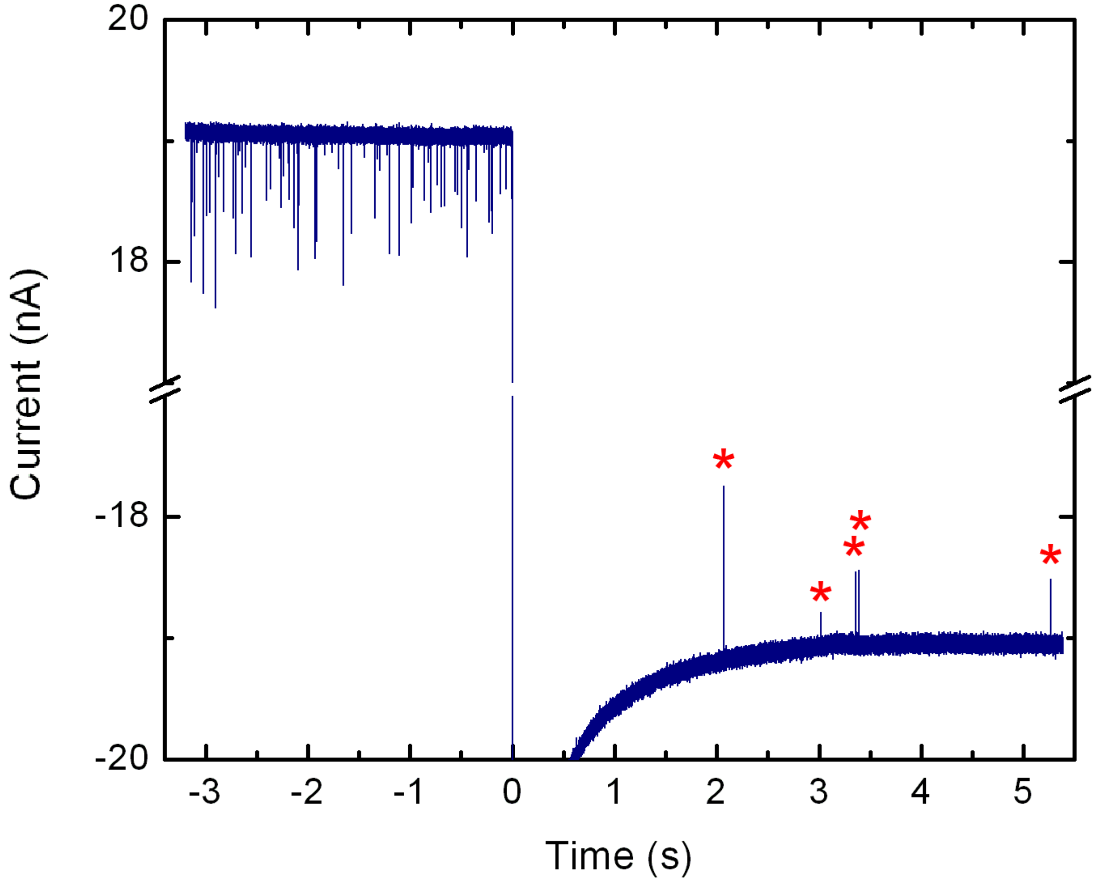

Supplementary Figure 1. Recapture of translocated HA Continuous current trace (400 mV/-400 mV) measured using polydisperse HA and a 6.5 nm SS-nanopore. After negative voltage is applied (*t*=0), recaptured HA events (*) are observed, confirming full translocation of material.

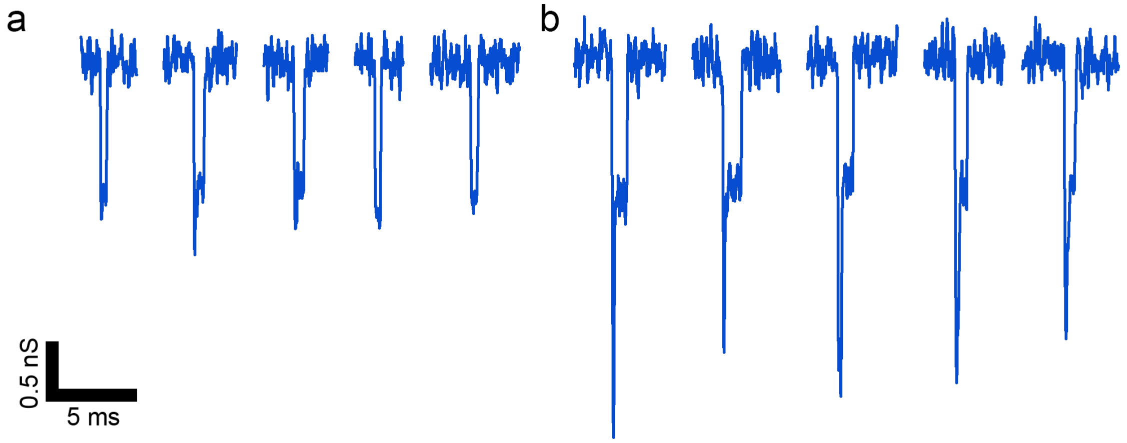

Supplementary Figure 2. Example individual HA translocation events Expanded view of typical conductance traces for 1,076 kDa quasi-monodisperse HA showing (a) unfolded molecules that maintain a single level for their duration and (b) folded molecules, marked by multiple conductance levels, the deeper of which is indicative of the folded portion. *Scale bar* applies to all traces.

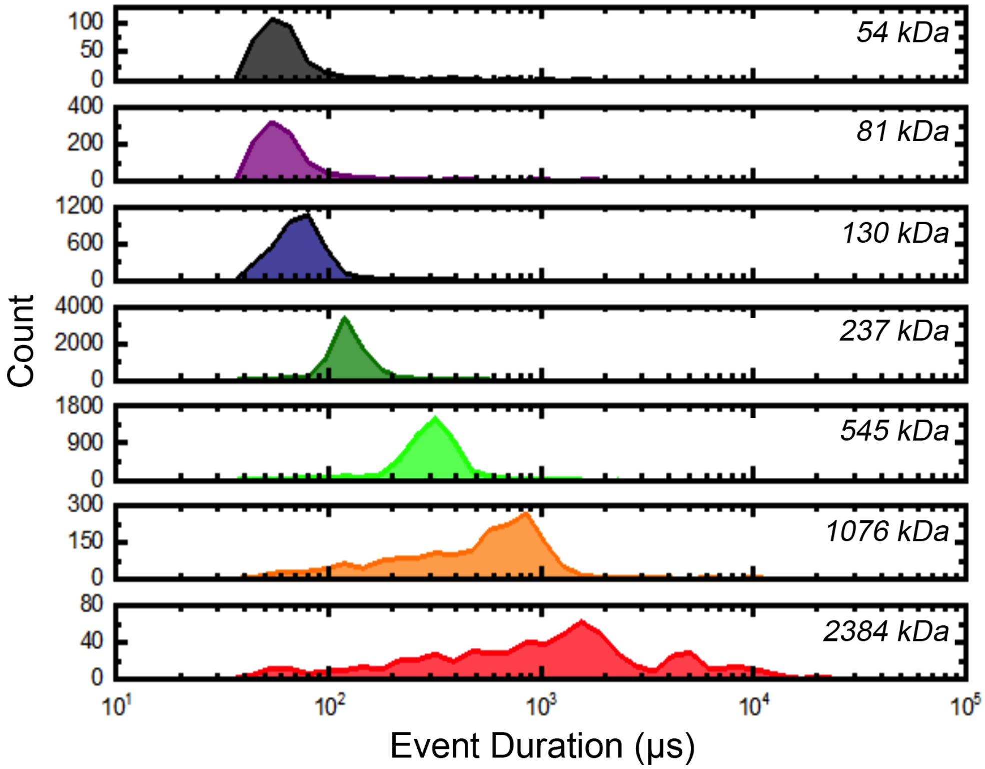

Supplementary Figure 3. Event duration analyses for quasi-monodisperse HA Event durations defined as the time the SS-nanopore current remains outside the 5σ threshold limit for a single translocation. Measurement was performed at 200 mV applied voltage. *N* values are the same as shown in **Fig. 3c-d**.

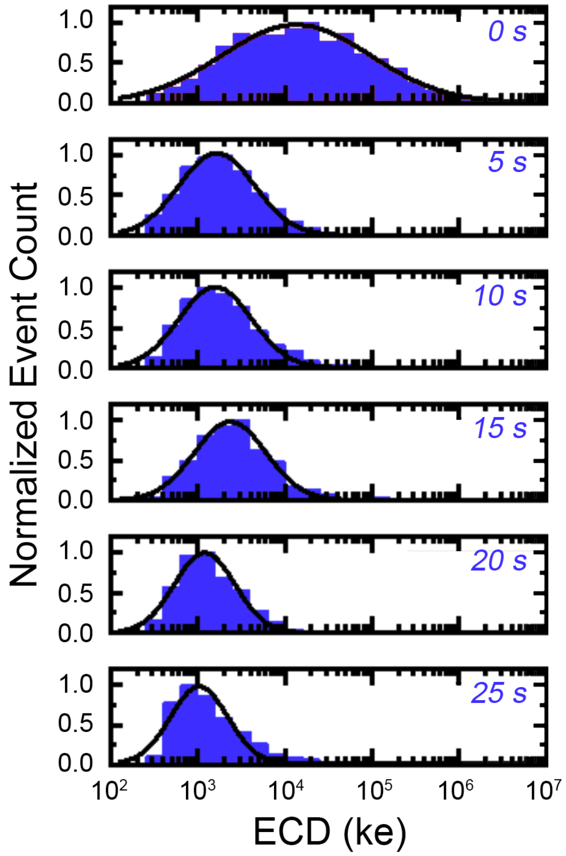

Supplementary Figure 4. ECD histograms for mechanically-sheared HA Individual ECD histograms for each set of sonicated polydisperse HA. The data for 0 s and 10 s samples are shown in **Fig. 2b** of the main text. *Black lines* are log-normal fits (Gaussian fits on a semi-log scale) to the data. *N* values are the same as shown in **Fig. 2b-c**. See **Supplementary Table 1** for fit details.

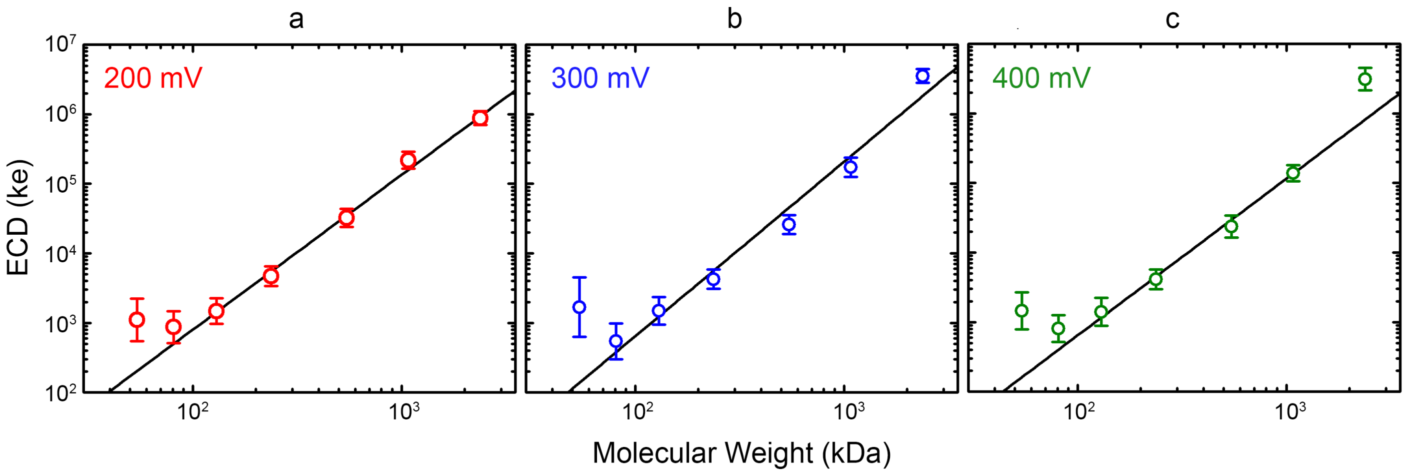

Supplementary Figure 5. Molecular weight dependence of ECD is conserved across voltages ECD vs. MW for quasi-monodisperse HA samples measured at 200 (*a*), 300 (*b*), and 400 mV (*c*). *Solid lines* are power-law fits to data down to 81 kDa (see **Supplementary Table 1** for fit details). *N* values are listed in **Supplementary Table 3** and error bars are standard deviations.

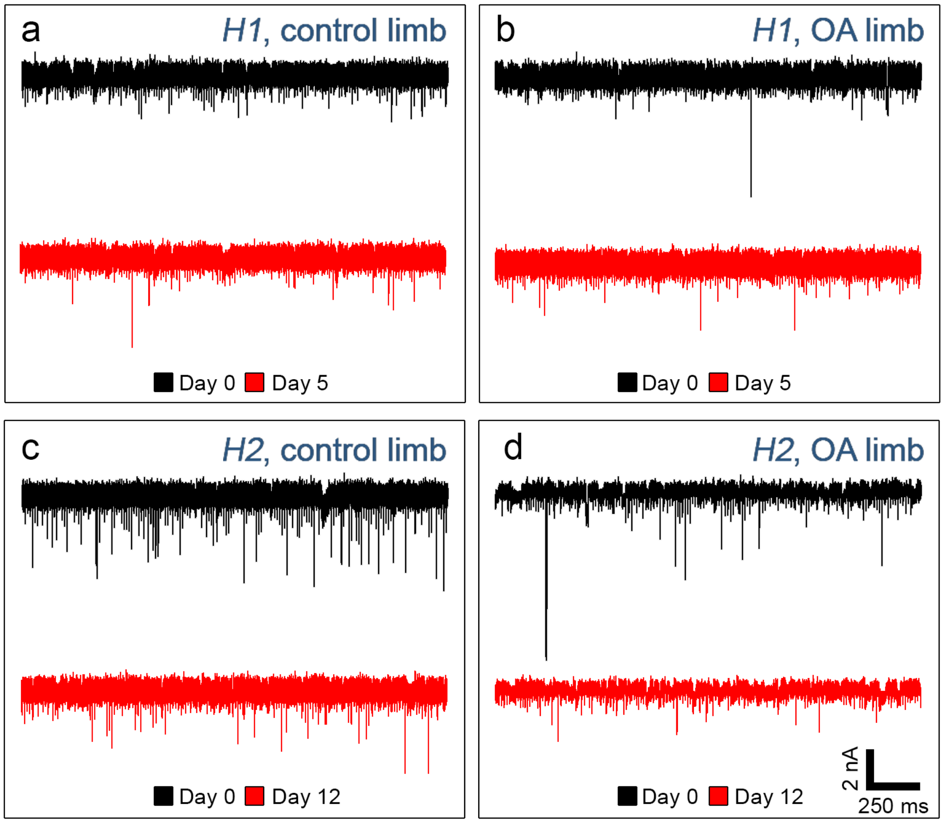

Supplementary Figure 6. Concatenated equine SF HA translocation events Example concatenated events collected for each horse, showing data from both sham limb (*a* and *c*) and OA limb (*b* and *d*) on Day 0 (pre-surgery) and Day 5 or Day 12 post-surgery. *Scale bar* applies to all traces.

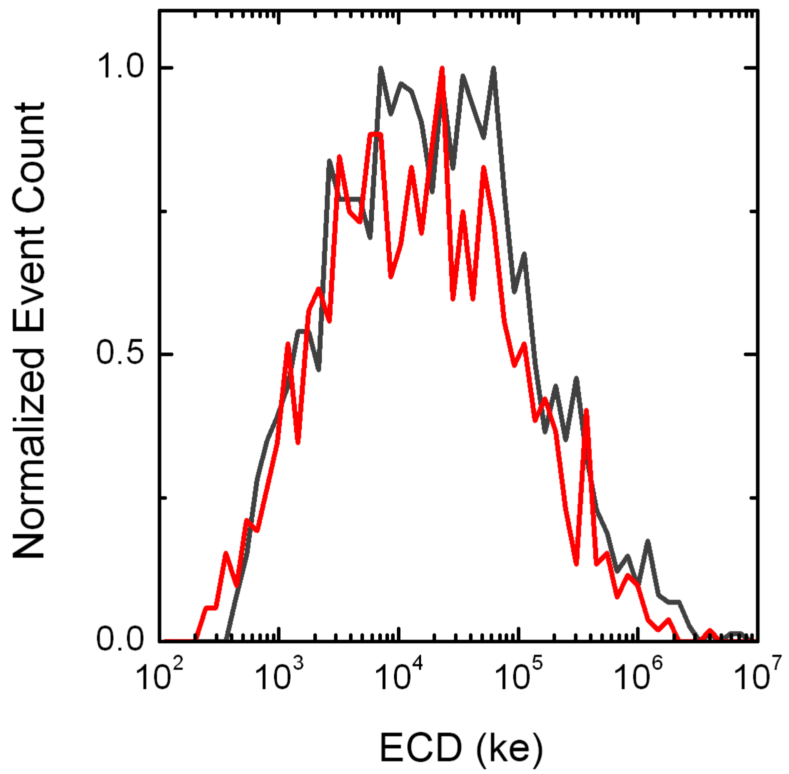

Supplementary Figure 7. Comparative polydisperse HA analysis ECD histograms obtained for polydisperse HA on two separate SS-nanopore devices. Diameters were 7.4 (*grey*, n=1775) and 6.2 nm (*red*, n=1067), respectively. Statistical analysis by t-test show the means are indistinguishable (p=0.39). Two-sample Kolmogorov-Smirnov (K-S) comparison indicate equivalent distributions (D=0.09 and exact p value is >0.05).

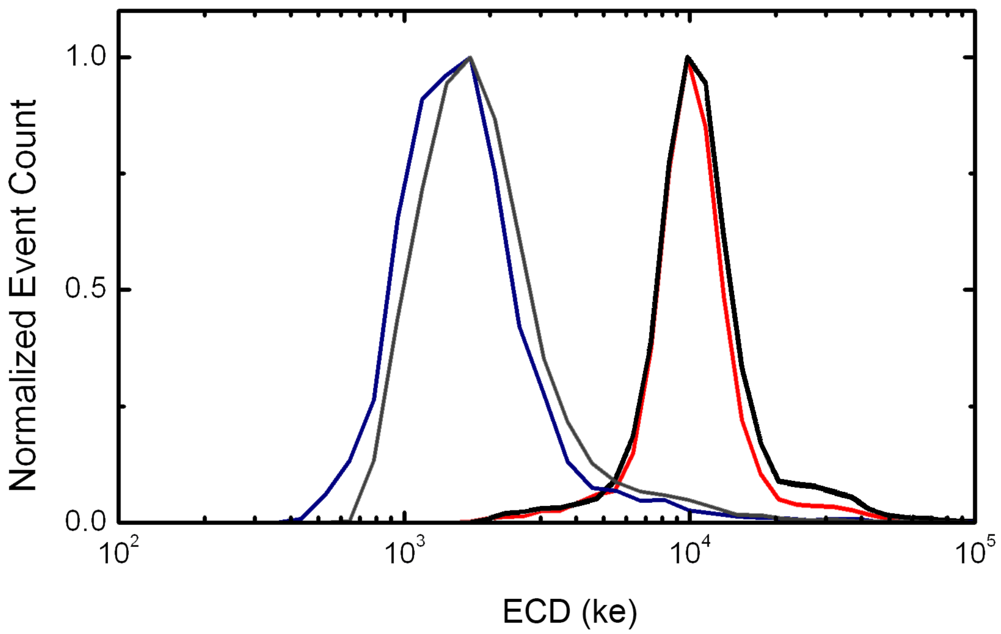

Supplementary Figure 8. Measurement consistency across distinct SS-nanopores ECD distributions obtained from two example quasi-monodisperse HA samples (*left*: 130 kDa, *right*: 237 kDa) measured on separate SS-nanopore devices. Pore diameters are 7.7 (*grey* and *black*), 8.6 (*blue*), 7.6 nm (*red*), respectively. Statistical analysis by t-test show the means for each set are indistinguishable (p=0.68 for 130 kDa; p=0.49 for 237 kDa). Two-sample K-S comparisons indicate equivalent distributions (D=0.14 for 130 kDa and D=0.10 for 237 kDa; exact p value is >0.05 for both). N values are 3667 (grey), 24379 (blue), 7835 (black), and 37378 (red).

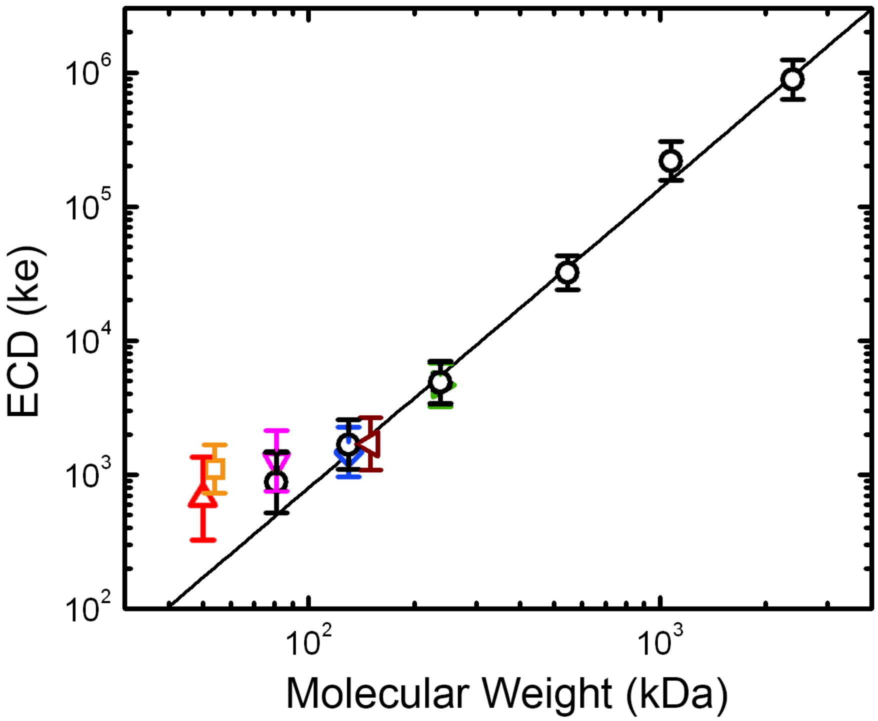

Supplementary Figure 9. ECD vs. HA MW on multiple SS-nanopore devices ECD as a function of MW measured on multiple distinct SS-nanopores. Pore diameters are 7.7 (*black*), 6.5 (*red*, membrane thickness 19 nm), 6.9 (*orange*), 7.4 (*magenta*), 8.6 (*blue*), and 8.4 nm (*maroon*, membrane thickness 19 nm), respectively. For *n* values, see **Supplementary Table 3**. *Solid line* is an exponential fit to the data down to 81 kDa (see **Supplementary Table 1** for fit details) and error bars are standard deviations.

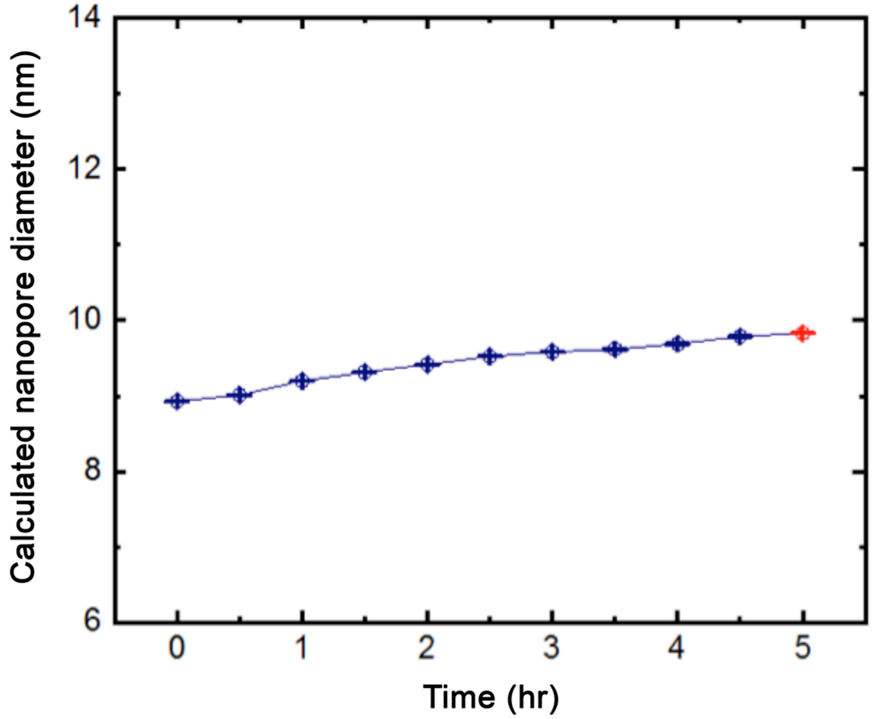

Supplementary Figure 10. SS-nanopore stability over time SS-nanopore diameter over a five hour time period under constant 200 mV applied voltage in measurement buffer. Diameter was calculated every 30 min from a linear I-V curve using an established analytical model[1] modified to incorporate empirical conductivity of high-concentration electrolyte in aqueous solution[2]. Each point is derived from an individual I-V measurement (taken from -200 to 200 mV) and error bars are the errors of the linear fits. *Red* data point was collected after replacing the buffer on both sides of the device to account for any effects of evaporation.

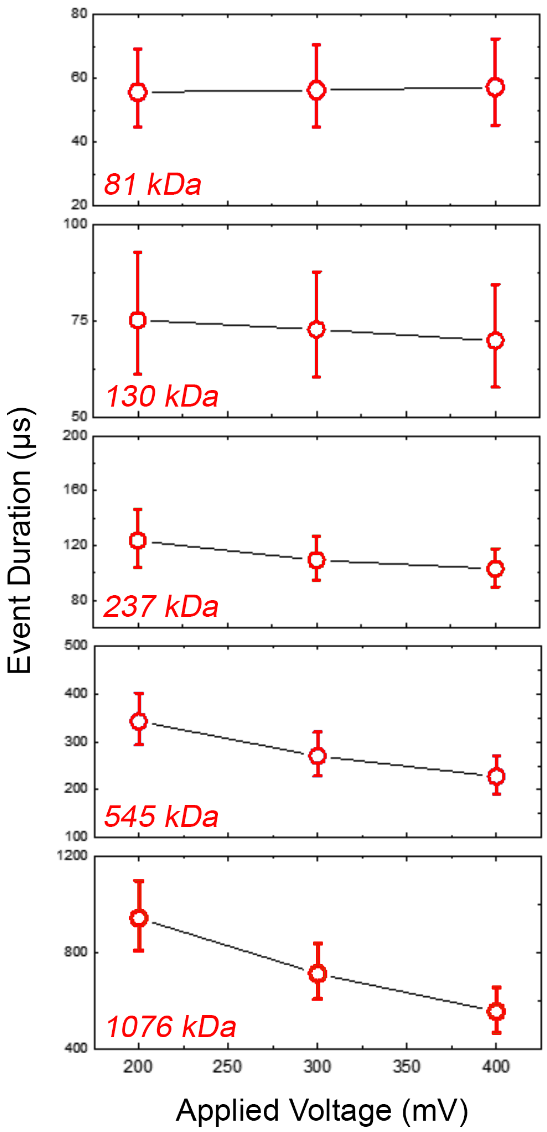

Supplementary Figure 11. Voltage dependence of quasi-monodisperse HA event durations Event durations from 200 to 400 mV applied voltage – determined as the time the SS-nanopore current remains outside the 5σ threshold limit – from 200 to 400 mV applied voltage using quasi-monodisperse HA across the range of 81-1,076 kDa. Only unfolded events are considered. Mean durations increase with MW (see **Supplementary Figure 3**) and thus reductions becomes more distinct. The largest MW sample (2,384 kDa) did not yield reliable duration result due to substantial molecular folding and significant fragmentation (c.f. **Fig. 3c** from main text). For *n* values, see **Supplementary Table 3**. Error bars are standard deviations.

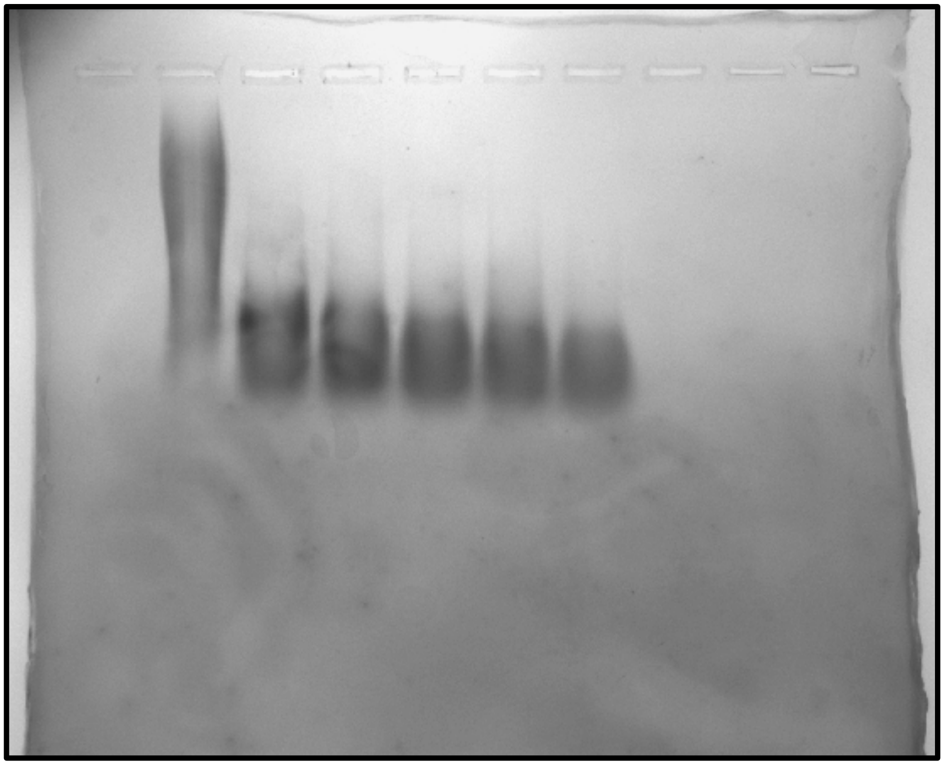

Supplementary Figure 12. Gel image of mechanically sheared HA Unprocessed image of gel showing sheared polydisperse HA shown in **Fig. 2a**.

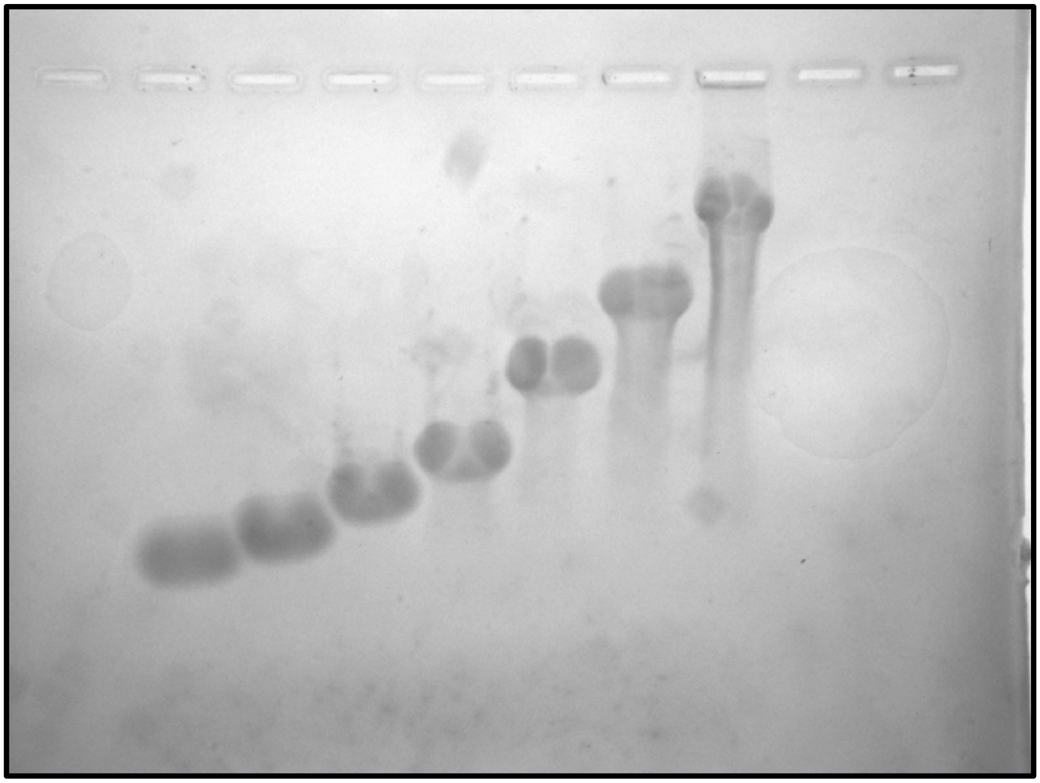

Supplementary Figure 13. Gel image of quasi-monodisperse HA Unprocessed image of gel showing quasi-monodisperse HA from **Fig. 3a**.

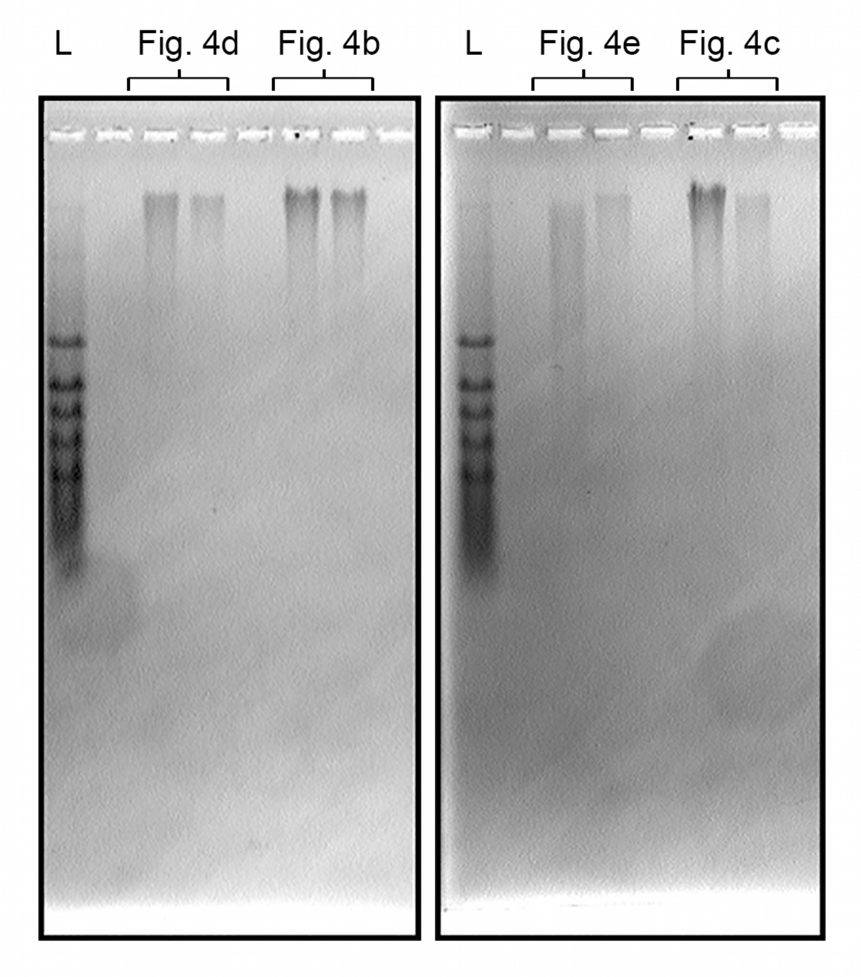

Supplementary Figure 14. Gel images of isolated equine synovial fluid HA Unprocessed images of gels showing physiological HA derived from equine synovial fluid. Positions in **Fig. 4** are indicated above. *L* indicates HA ladder.

Measurement	Fit equation	Data location	Fit parameters		Best fit values	±Error
Polydisperse HA sonication (fit on a semi-log scale)	$y=\frac{A}{w\sqrt{\pi/2}}e^{-2\frac{(x-x_c)^2}{w^2}}$	Fig. 2b (red) Supp. Fig. 4 (0 s)	A	area	2.08	0.06
			w	width	1.70	0.06
			x_c	center	4.12	0.03
		Supp. Fig. 4 (5 s)	A	area	1.12	0.02
			w	width	0.87	0.02
			x_c	center	3.22	0.01
		Fig. 2b (blue) Supp. Fig. 4 (10 s)	A	area	1.05	0.03
			w	width	0.83	0.03
			x_c	center	3.20	0.02
		Supp. Fig. 4 (15 s)	A	area	1.01	0.03
			w	width	0.83	0.02
			x_c	center	3.37	0.01
		Supp. Fig. 4 (20 s)	A	area	0.87	0.03
			w	width	0.70	0.03
			x_c	center	3.08	0.01
		Supp. Fig. 4 (25 s)	A	area	0.83	0.05
			w	width	0.68	0.05
			x_c	center	3.02	0.02
Quasi-monodisperse samples (fit on a semi-log scale)	$y=\frac{A}{w\sqrt{\pi/2}}e^{-2\frac{(x-x_c)^2}{w^2}}$	Fig. 3c (black)	A	area	0.45	0.014
			w	width	0.62	0.013
			x_c	center	3.04	0.006
		Fig. 3c (purple)	A	area	0.53	0,021
			w	width	0.46	0.021
			x_c	center	2.94	0.010
		Fig. 3c (blue)	A	area	0.49	0.005
			w	width	0.37	0.005
			x_c	center	3.17	0.003
		Fig. 3c (dark green)	A	area	0.34	0.004
			w	width	0.28	0.004
			x_c	center	3.67	0.002
		Fig. 3c (green)	A	area	0.33	0.005
			w	width	0.26	0.005
			x_c	center	4.51	0.002
		Fig. 3c, (orange)	A	area	0.27	0.007
			w	width	0.25	0.008
			x_c	center	5.34	0.003
		Fig. 3c (red)	A	area	0.20	0.016
			w	width	0.20	0.016
			x_c	center	5.95	0.006
Quasi-monodisperse ECD vs. MW Power law fit	$y=bx^{\alpha}$	Fig. 3d; Supp. Fig. 5a	b	scaling	0.028	0.016
			α	exponent	2.228	0.084
		Supp. Fig. 5b	b	scaling	0.006	0.011
			α	exponent	2.509	0.285
		Supp. Fig. 5c	b	scaling	0.020	0.029
			α	exponent	2.254	0.237

Supplementary Table 1. Fit details Parameters and results for log-normal (Gaussian on a semi-log scale) and power law fits of all data.

Sample	Mean apparent HA concentration (ng/ml)*	Dilution factor	Actual concentration (ng/µl)	Solution volume (µl)	Total HA mass (ng)	Mean HA mass (ng)
R1	719.7	500	359.8	100	35,984	39,128 ± 4,973
R2	365.4	1000	365.4	100	36,539	
R3	224.3	2000	448.6	100	44,862	
PC1	634.9	500	317.4	100	31,743	35,211 ± 3,004
PC2	370.2	1000	370.2	100	37,022	
PC3	184.3	2000	368.7	100	36,868	
BE1	512.1	7.8	4.0	50	200	159 ± 59
BE2	142.0	16.5	2.3	50	117	

Supplementary Table 2. ELISA results for isolation protocol steps Determination of HA abundance throughout isolation procedure for a typical equine synovial sample. **R** is raw synovial fluid, **PC** is the same sample post-phenol/chloroform treatment, and **BE** (bead elution) is HA amount recovered from magnetic beads, suitable for direct SS-nanopore analysis at the end of the process. Multiple dilutions were analyzed for each sample. *<10% systematic error is associated with ELISA.

Figure	Data Description	*n* values
Fig. 1d (*n* = number of uninterrupted 3.2 s current traces considered)	5 ng/μl	*l-r:* 167, 171, 171, 200, 169, 168
	10 ng/μl	*l-r:* 127, 131, 132, 132, 132, 132
	25 ng/μl	*l-r:* 60, 64, 62, 58, 66, 72
	50 ng/μl	*l-r:* 26, 26, 26, 27, 26, 26
	75 ng/μl	*l-r:* 28, 32, 28, 29, 33, 30
Supp. Fig. 5	200 mV	*l-r:* 344, 1031, 3667, 7835, 5012, 1743, 640
	300 mV	*l-r:* 1196, 9988, 12228, 12187, 9199, 5790, 548
	400 mV	*l-r:* 1985, 9746, 16320, 10927, 7193, 3107, 471
Supp. Fig. 9	*black*	*l-r:* 1031, 3667, 7835, 5012, 1743, 640
	red	19297
	orange	344
	magenta	1504
	blue	24379
	maroon	1684
	green	37378
Supp. Fig. 11	81 kDa	200 mV: 708, 300 mV: 3254, 400 mV: 9862
	130 kDa	200 mV: 2758, 300 mV: 18378, 400 mV: 19311
	237 kDa	200 mV: 5392, 300 mV: 11153, 400 mV: 13107
	545 kDa	200 mV: 2676, 300 mV: 4282, 400 mV: 5993
	1076 kDa	200 mV: 843, 300 mV: 3009, 400 mV: 2835

Supplementary Table 3. *N* values for display data Number of discrete traces (for Fig. 1d) or translocation events (all others) considered for displayed data in indicated Figures.

Supplementary References

1. Wanunu, M., Dadosh, T., Ray, V., Jin, J., McReynolds, L. & Drndic, M. Rapid electronic detection of probe-specific microRNAs using thin nanopore sensors. *Nat. Nanotechnol.* **5,** 807–814 (2010).

2. Haynes, W. M. *CRC Handbook of Chemistry and Physics, 97th Edition*. (CRC Press, Boca Raton, FL, 2016)
